# The association between diabetes status and latent-TB IGRA levels from a cross-sectional study in eastern China

**DOI:** 10.3389/fcimb.2022.1057298

**Published:** 2023-01-16

**Authors:** Cheng Chen, Xinsong Hu, Yan Shao, Honghuan Song, Guoli Li, Wei Lu, Leonardo Martinez, Jianfang Xu, Limei Zhu

**Affiliations:** ^1^ Department of Chronic Communicable Disease, Center for Disease Control and Prevention of Jiangsu Province, Nanjing, China; ^2^ Department of Epidemiology and Statistics, School of Public Health, Nanjing Medical University, Nanjing, China; ^3^ Department of Epidemiology, School of Public Health, Boston University, Boston, MA, United States; ^4^ Department of Infectious Disease Control, Center for Disease Control and Prevention of Danyang County, Zhenjiang, China

**Keywords:** tuberculosis, interferon gamma, diabetes, latent tuberculosis infection, cross-sectional study

## Abstract

**Background:**

There is a debate regarding the sensitivity of the QuantiFERON-TB Gold In-Tube (QFT) among people with diabetes, and prior studies have shown heterogeneous results. We evaluated whether the QFT TB antigen was modified among persons with differing diabetes status and other related risk factors.

**Methods:**

A cross-sectional study of 5,302 people was conducted to screen latent tuberculosis infection (LTBI) in eastern China. The QFT assay was performed as an indicator of LTBI. Fasting plasma glucose (FPG) was collected from each participant; the definition of diabetes followed the guidelines from the American Diabetes Association. Participants were classified into normoglycemia, prediabetes, undiagnosed diabetes, and previously diagnosed diabetes to evaluate the relationship between the QFT TB antigen and distinct diabetes status.

**Results:**

TB antigen values from the QFT were statistically different among participants with differing diabetes status (*P* = 0.008). Persons with undiagnosed diabetes had a higher TB antigen value (0.96 ± 0.20) than persons with normoglycemia (0.50 ± 0.02, *P* < 0.05). However, the TB antigen values demonstrated no significant difference among the four different diabetic groups when stratified by the standard cutoff for the QFT (*P* = 0.492 for the positive group and *P* = 0.368 for the negative group). In a linear regression model, we found that FPG, age, and smoking were positively associated with the QFT TB antigen value (*P* = 0.017, *P* < 0.001, and *P* < 0.001).

**Conclusions:**

Diabetes status had little influence on the level of QFT TB antigen response among IGRA-positive persons. However, FPG, old age, and smoking were important risk factors for increasing levels of QFT TB antigen.

## Introduction

It was estimated that one-quarter of the global population was infected by *Mycobacterium* tuberculosis (Cohen et al., 2019). Currently, the tuberculin skin test (TST) and interferon-gamma release assay (IGRA)-based methods are widely used to detect latent tuberculosis infection (LTBI). However, there are some disadvantages of TST, such as misclassification in settings with high BCG vaccination coverage and failing to differentiate non-tuberculous infection (Farhat et al., 2006). Nevertheless, due to its low cost, the TST is still considered an important alternative for LTBI screening in resource-limited regions. IGRA-based methods have a higher specificity than the TST (Campbell et al., 2015). However, limitations include the potential for indeterminate results and debate over the correct cutoff to indicate positivity. Moreover, IGRA-based methods are not recommended for certain populations, such as patients receiving immunosuppressive treatment (Kobashi et al., 2007).

The diabetes disease burden has dramatically increased globally in recent years. Low- and middle-income countries, which have the largest burden of tuberculosis, have seen the greatest growth in diabetes (Liu et al., 2022). The number of persons with diabetes is estimated to be approximately 100 million in 2025 in China (Zhu et al., 2021). Several studies have suggested that diabetes may increase the risk of latent tuberculosis infection (Lee et al., 2017; Barron et al., 2018). Diabetes may impact an individual’s immunological response. Several studies had debated on whether the sensitivity of the QuantiFERON-TB Gold In-Tube (QFT) was distinct or not among persons with diabetes (Walsh et al., 2011; Choi et al., 2015). We aimed to investigate whether persons with diabetes have distinct responses to the IGRA test in a rural Chinese setting with high rates of BCG vaccination. In our study, we evaluated whether the TB antigen level of the QFT was modified based on fasting plasma glucose (FPG) levels in a general population.

## Methods

### Study design and population

**Figure 1:**
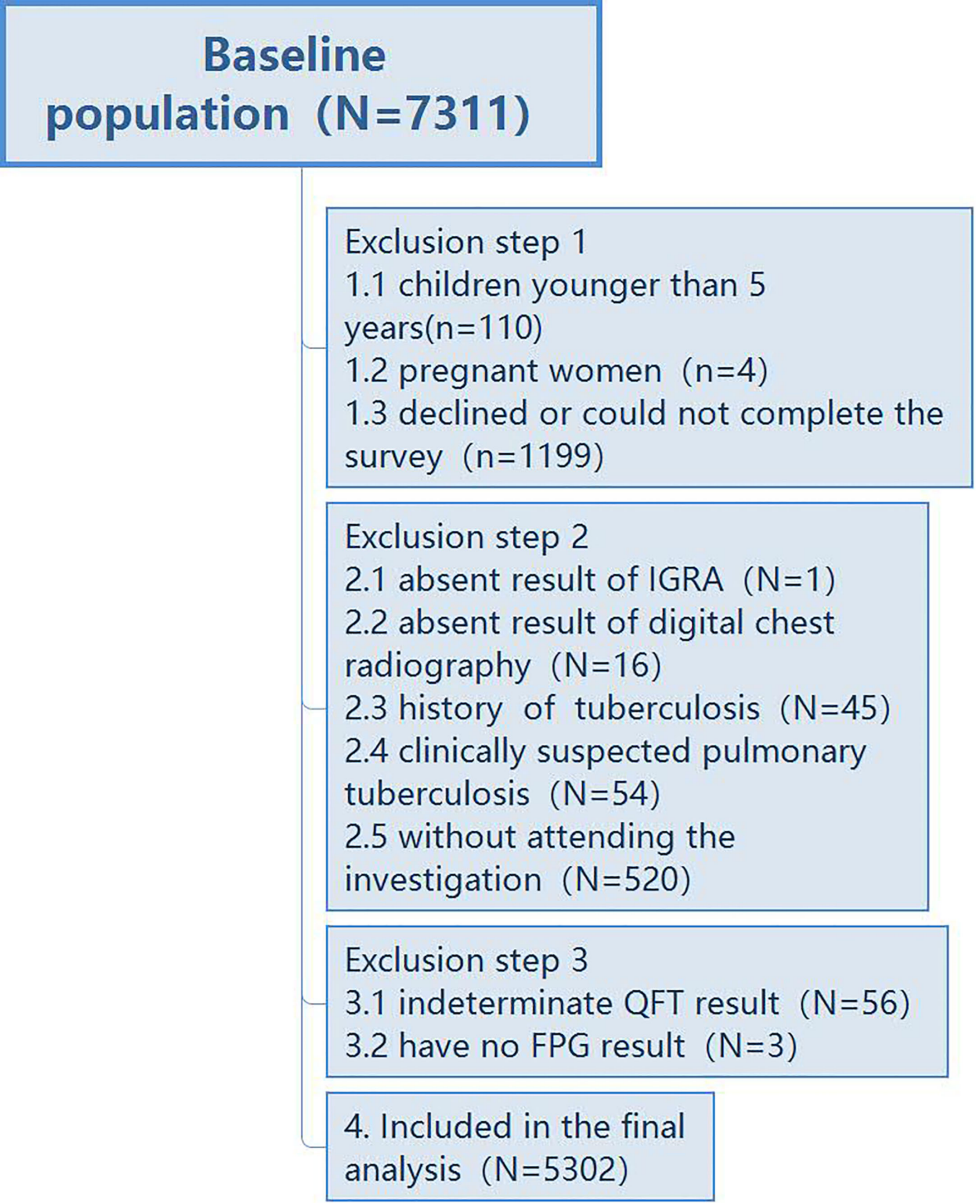
Flowchart of the inclusion of the study population.

Between 01/07/2013 and 31/07/2013, a cross-sectional study on LTBI was conducted in two villages of Danyang County, Jiangsu Province. People with a residence time more than 6 months were screened, and the residence population was 7,311. In the household investigation to know people who want to participate in the screening, 1,199 people declined to participate in the study; 110 children aged less than 5 years and 4 pregnant women were excluded. In the second step, questionnaires and health examination were conducted on all eligible participants in the clinics. Several exclusions occurred including 54 persons with clinically suspected pulmonary TB, 45 persons with a history of TB, 16 persons who did not have chest radiography, one person who did not take the IGRA test, and 520 people who did not attend the examination. In addition, 56 persons with an indeterminate IGRA result and three persons without an FPG result were also excluded. In total, 5,302 participants were included (**Figure 1**). The local CDC registered those new TB cases diagnosed by the local TB hospital from this active screening. The study was approved by the ethics committee of the Institute of Pathogen Biology, Chinese Academy of Medical Sciences, and all participants provided written informed consent before the investigation.

### Measures and definitions

Diabetes was defined based on the baseline fasting plasma glucose (FPG) levels according to American Diabetes Association guidelines (American Diabetes, 2020). The study population was classified as normoglycemia (FPG <6.1 mmol/l), prediabetes (pre-DM: FPG from 6.1 to 7.0 mmol/l), undiagnosed diabetes (FPG >7.0 mmol/l and without a previous diagnosis of diabetes), and diagnosed diabetes among persons that had been diagnosed with diabetes previously. LTBI status of participants was determined by the QuantiFERON-TB Gold In-Tube (QFT) test, and procedures for administration of the QFT can be found in prior work on this cohort (Whitworth et al., 2013). 5% of the samples were randomly selected to repeat for consistency, and all results were 100% consistent with the primary results.

### Data analysis

We compared between-group demographics using Pearson’s χ^2^ test or Fisher exact tests for categorical data. Unpaired Student’s *t-*test was applied between two groups for continuous variables, and one-way ANOVA tests were used to compare continuous variables for more than two groups, whereas Dunnett’s *t* test was employed to analyze the variance of subgroups compared with the control group where ANOVA showed a significant difference among all subgroups. We conducted a linear regression model to measure the relationship between TB antigen and potential risk factors. *P* values less than 0.05 were considered statistical significance. All analyses were performed using SAS 9.3 software (SAS Institute, Inc., Cary, NC, USA).

## Results

The total population included in this survey was 5,361. Among these participants, 56 QFT results were indeterminate and three participants did not have an FPG result. In total, 5,302 people were included in the final analyses. People with a positive QFT were more likely to be older (57.51 ± 13.10 years versus 48.07 ± 18.02 years, *P* < 0.001) and had a higher body mass index (BMI: 23.75 ± 3.24 versus 23.23 ± 3.66, *P* < 0.001). LTBI was also disproportionated among sex, smoking, and drinking status, respectively (*P* < 0.001, *P* < 0.001, *P* < 0.001). Moreover, we found that the proportion of diabetes (both previously diagnosed diabetes and undiagnosed diabetes) in LTBI was higher than in those that were QFT negative (7.5% *vs*. 5.1%); persons with a positive QFT were also more likely to have a higher FPG than persons testing negative (5.07 ± 1.57 *vs*. 4.94 ± 1.38 mmol/l, *P* = 0.007) (**Table 1**).

**Table 1:** Comparison of baseline characteristics between QFT negative and QFT positive populations.

Characteristic	Total (N=5302)	Negative QFT (N=4243)	Positive QFT (N=1059)	χ^2^	*P* value
Age				219.391	<0.001
Mean ± SD (years)	49.96 ± 17.57	48.07 ± 18.02	57.51 ± 13.10		<0.001^*^
<20	417 (7.9%)	410 (9.7%)	7 (0.7%)		
20-40	880 (16.6%)	794 (18.7%)	86 (8.1%)		
40-60	2452 (46.2%)	1930 (45.5%)	522 (49.8%)		
>60	1553 (29.3%)	1109 (26.1%)	444 (41.9%)		
Sex				37.107	<0.001
Male	2466 (46.5%)	1885 (44.4%)	581 (54.9%)		
Female	2836 (53.5%)	1358 (55.6%)	479 (45.1%)		
Smoking				74.912	<0.001
No	4011 (75.7%)	3318 (78.2%)	693 (65.4%)		
Yes	1291 (24.3%)	925 (21.8%)	366 (34.6%)		
Drinking				43.855	<0.001
No	4117 (77.6%)	3375 (79.5%)	742 (70.1%)		
Yes	1185 (22.4%)	868 (20.5%)	317 (29.9%)		
Body mass index				20.031	<0.001
Mean ± SD	23.34 ± 3.58	23.23 ± 3.66	23.75 ± 3.24		<0.001^*^
<18.5	330 (6.2%)	289 (6.8%)	41 (3.9%)		
18.5-24	2884 (54.4%)	2331 (54.9%)	553 (52.2%)		
24-28	1641 (31.0%)	1272 (30.0%)	369 (34.8%)		
>28	447 (8.4%)	351 (8.3%)	96 (9.1%)		
Glucose status				11.947	0.008
No diabetes	4869 (91.8%)	3915 (92.3%)	954 (90.1%)		
Pre- diabetes	138 (2.6%)	112 (2.6%)	26 (2.5%)		
Previously diagnosed diabetes	117 (2.2%)	80 (1.9%)	37 (3.5%)		
Undiagnosed diabetes	178 (3.4%)	136 (3.2%)	42 (4.0%)		
FPG Mean ± SD (mmol/L)	4.96 ± 1.42	4.94 ± 1.38	5.07 ± 1.57		0.007^*^

^*^Unpaired student t test for continuous variables among QFT position and negative groups.

FPG: fasting plasma glucose

The TB antigen values of the QFT were significantly different among persons with distinct glucose levels (*P* = 0.008, **Table 2**). Persons with undiagnosed diabetes had higher TB antigen values (0.96 ± 0.20) compared with persons who had normoglycemia (0.50 ± 0.02, *P* < 0.05). The level of TB antigen was highest among persons with undiagnosed diabetes. When stratifying by age, sex, smoking, and drinking, compared with the normoglycemia group, we found that the level of TB antigen was significantly higher for undiagnosed diabetes among those aged between 40 and 60 years (1.22 ± 0.29 IU/ml, *P* < 0.05), those women (0.95 ± 0.32 IU/ml, *P* < 0.05), non-smokers (0.95 ± 0.25 IU/ml, *P* < 0.05), and non-drinkers (0.89 ± 0.24 IU/ml, *P* < 0.05). However, the TB antigen values among the four different diabetic groups demonstrated no significant difference when stratifying by the QFT-positive group (*P* = 0.492) and QFT-negative group (*P* = 0.368).

**Table 2:** TB antigen values among different diabetic groups.

Populations	N	Group 1: normoglycemia (TB antigen, IU/mL)	N	Group 2: prediabetes (TB antigen, IU/mL)	N	Group 3: undiagnosed diabetes (TB antigen, IU/mL)	N	Group 4: previously diagnosed diabetes (TB antigen, IU/mL)	*P* value for the four-group comparisons
Total population	n = 4,869	0. 50 ± 0.02	138	0.57 ± 0.14	117	0.96 ± 0.20^*^	178	0.63 ± 0.13	0.008
Age (years)									
<20	n = 416	0.04 ± 0.01	n = 1	0.04	n = 0	NA	n = 0	NA	0.761
≥20 and <40	n = 867	0.27 ± 0.04	n = 3	0.60 ± 0.59	n = 8	0.23 ± 0.19	n = 2	0.04 ± 0.04	0.938
≥40 and <60	n = 2,235	0.58 ± 0.03	n = 66	0.60 ± 0.23	n = 74	1.22 ± 0.29^*^	n = 77	0.40 ± 0.22	0.009
≥60	n = 1,351	0.65 ± 0.04	n = 68	0.55 ± 0.17	n = 35	0.58 ± 0.28	n = 99	0.75 ± 0.18	0.872
Sex									
Male	n = 2,286	0.56 ± 0.03	n = 64	0.91 ± 0.27	n = 60	0.97 ± 0.26	n = 56	1.12 ± 0.33^*^	0.004
Female	n = 2,583	0.44 ± 0.03	n = 74	0.28 ± 0.09	n = 57	0.95 ± 0.32^*^	n = 122	0.34 ± 0.13	0.029
Smoking									
No	n = 3,673	0.42 ± 0.02	n = 101	0.35 ± 0.10	n = 84	0.95 ± 0.25^*^	n = 153	0.46 ± 0.13	0.004
Yes	n = 1,196	0.74 ± 0.05	n = 37	0.18 ± 0.43	n = 33	1.00 ± 0.35	n = 25	1.37 ± 0.59	0.158
Drinking									
No	n = 3,783	0.45 ± 0.02	n = 99	0.37 ± 0.10	n = 80	0.89 ± 0.24^*^	n = 155	0.46 ± 0.13	0.047
Yes	n = 1,086	0.65 ± 0.05	n = 39	1.09 ± 0.41	n = 37	1.12 ± 0.39	n = 23	1.45 ± 0.64	0.026
QFT positive population	n = 954	2.36 ± 0.08	n = 26	2.80 ± 0.55	n = 37	2.92 ± 0.51	n = 42	2.50 ± 0.45	0.492
QTF negative population	n = 3,915	0.04 ± 0.01	n = 112	0.05 ± 0.01	n = 80	0.06 ± 0.01	n = 136	0.06 ± 0.01	0.368

^*^P < 0.05 for TB antigen values among groups 2, 3, and 4 when compared with group 1, respectively; NA, not available.

The linear regression plot for TB antigen values and FPG is shown in **Figure S1** (Supplementary), and we further analyzed the QFT TB antigen values in relation to different factors and distinct populations. In the entire study population, increased age and smoking were positively associated with the increased level of QFT TB antigen value (*P <* 0.001 and *P* < 0.001) but the FPG was not found in association with QFT antigen value (*P* = 0.147) (**Table 3**). Persons with previously diagnosed diabetes persons might have been given interventions like glucose control treatment or physical activity interventions. Therefore, in a secondary analysis we excluded previously diagnosed diabetes from the analysis. We found that increased FPG, old age, and smoking were positively associated with increased QFT TB antigen values (*P* = 0.023, *P* < 0.001, and *P* < 0.001).

**Table 3:** Linear regression models for QFT TB antigen (IU/mL) and associated factors among different populations.

QFT TB antigen (IU/mL)	Variables	β-estimates for FPG	Standard error	*P*
QFT TB antigen for all populations	Intercept	-0.2543	0.1326	0.055
	FPG	0.0210	0.0145	0.147
	age	0.1684	0.0243	<0.001
	sex	-0.0110	0.0529	0.836
	BMI	0.0437	0.0283	0.124
	smoking	0.2703	0.0615	<0.001
	drinking	0.0344	0.0594	0.562
QFT TB antigen for subgroup: (normal glucose, prediabetes, and undiagnosed diabetes)	Intercept	-0.3240	0.1409	0.022
	FPG	0.0405	0.0178	0.023
	age	0.1680	0.0243	<0.001
	sex	0.0071	0.0532	0.894
	BMI	0.0240	0.0287	0.402
	smoking	0.2751	0.0617	<0.001
	drinking	0.0218	0.0596	0.715
QFT TB antigen for subgroup with best model: (normal glucose, prediabetes, and undiagnosed diabetes)	Intercept	-0.2740	0.1048	0.009
	FPG	0.0422	0.0177	0.017
	age	0.1725	0.0236	<0.001
	smoking	0.2818	0.0478	<0.001

FPG, fasting plasma glucose.

Age, sex, BMI, smoking, and drinking were category data, which are defined in **Table 1**.

## Discussion

In this large-scale cross-sectional study, characteristics such as old age, male sex, smoking, drinking, diabetes, high BMI, and high glucose level were associated with LTBI. Furthermore, even though we found that QFT TB antigen values significantly increased among persons with undiagnosed diabetes compared with the normoglycemia group, we found that, upon stratification of QFT results, QFT TB antigen values were not significantly increased from the normoglycemia group to the undiagnosed diabetes population, indicating that diabetes status had little influence on the prevalence of QFT positivity. The linear regression model demonstrated that FPG, age, and smoking were independently associated with QFT TB antigen and they could be important indicators for LTBI exposure.

WHO reported that systematic testing for LTBI was not recommended for people with diabetes, but evidence for this recommendation was considered of low quality (Anonymous, 2018). For the diabetic population, the impaired immune responses may increase the susceptibility to tuberculosis infection (Vallerskog et al., 2010; Gomez et al., 2013). Diabetes was associated with an increased prevalence of LTBI in prior studies (Barron et al., 2018). A previous systematic review and meta-analysis showed that diabetes was associated with a small increased risk of LTBI (Lee et al., 2017), but a recent meta-analysis showed that the risk of LTBI was found to be a 60% increased risk in persons with diabetes and a 36% increased risk in prediabetes when compared with normoglycemia people (Liu et al., 2022). In addition, there were studies indicating the increased risk of incident diabetes among individuals with LTBI (Magee et al., 2022), demonstrating that the LTBI population was a vulnerable group of people who were susceptible to diabetes.

We assessed whether the increased prevalence of LTBI among person with diabetes was present among the sample tested with IGRAs. As we used an indirect method of testing LTBI, whether the positive response of IGRA was modified by the glucose level was unknown for the general population. A previous study focused on IGRA response between diabetes and non-diabetes tuberculosis patients and found no significant differences in the qualitative or quantitative IGRA responses to *Mycobacterium* tuberculosis-specific antigens as measured by the QFT and T-SPOT.TB assays (Gan et al., 2014). Choi et al. revealed a reduced sensitivity of QFT among diabetic TB patients (Choi et al., 2015). Therefore, LTBI detection among TB patients was debated, even though LTBI detection was not necessary for tuberculosis diagnosis.

However, there has been no study on diabetes status impacting LTBI detection from the general population. Our study evaluated QFT TB antigen among normal glucose people, prediabetes, unknown diabetes, and previously diagnosed diabetes. We found an increasing level of TB antigen from persons with normoglycemia compared with those with previously diagnosed diabetes. The increased difference for QFT TB antigen was only observed between normoglycemia people and undiagnosed diabetes population. The difference might be induced by a high proportion of positive QFT among undiagnosed diabetes than in the other glucose level groups. However, the trend of increasing difference could be observed from either the QFT-positive or negative group, even though the difference reached no statistical difference, which might be due to small samples for prediabetes, undiagnosed diabetes, and those previously diagnosed diabetes.

Our study also indicated that the previously diagnosed diabetes population seemed to have a lower QFT TB antigen than the undiagnosed diabetes population, partly because the previously diagnosed diabetes population was receiving a treatment of diabetes and a change to good living habits after the diagnosis of diabetes. Thus, glucose control intervention after the diagnosis of diabetes might modify the response of the QFT TB antigen (Martinez et al., 2017).

In the linear regression model for QFT TB antigen and FPG, it was found that FPG was independently associated with QFT TB antigen, suggesting that the high QFT TB antigen was correlated with high FPG or diabetic status. On the contrary, diabetes was associated with an increased risk of LTBI. There was also a challenge for LTBI detection among the diabetes population because a high indeterminate result for QFT was found in those diabetic TB patients than in normoglycemia TB patients (Gan et al., 2014).

In this population-based study, we also found that old age and smoking were associated with an increased value of QFT TB antigen, implying that old age and smoking might be associated with an increased risk of LTBI. Previous studies found that smoking was associated with a high risk of LTBI (Horne et al., 2012; Lindsay et al., 2014) and that secondhand smoking exposure was also considered a factor related to LTBI (Altet et al., 2022; du Preez et al., 2011). Meanwhile, smoking quantity and IFN-γ response were dose-dependently related for smoking contacts, which implied that smoking contacts with a negative LTBI should be careful of interpretation of LTBI status (Altet et al., 2017). A recent review demonstrated that smoking impaired innate barrier defense, as well as alveolar macrophage, neutrophil, dendritic cell, and T-cell functions, in the context of TB infection and disease (Quan et al., 2022).

There are several limitations to this study. Firstly, this was a cross-sectional study and whether glucose levels could modify IGRA responses should be validated in prospective, longitudinal studies. Second, treatment information of previously diagnosed diabetes was not available. Third, there were small sample sizes among people with prediabetes and undiagnosed diabetes. Larger sample sizes among these groups are warranted in future studies to verify the associations we found in this study. In conclusion, we found that QFT TB antigen values significantly increased among persons with undiagnosed diabetes compared with the normoglycemia group. Despite this, diabetes status had little influence on the detection of LTBI. Continuously, FPG value, old age, and smoking were risk factors for increasing levels of QFT TB antigen.

## Data availability statement

The raw data supporting the conclusions of this article will be made available by the authors, without undue reservation.

## Ethics statement

The studies involving human participants were reviewed and approved by the ethics committee of the Institute of Pathogen Biology, Chinese Academy of Medical Sciences. Written informed consent to participate in this study was provided by the participants’ legal guardian/next of kin.

## Author contributions

CC and XH wrote the draft of the manuscript. LM, JX, and LZ designed and edited the manuscript. YS, HS, and GL conducted the experiments. WL, LZ, and CC reviewed the data collection. All authors contributed to the article and approved the submitted version.

## Supplementary material

Supplementary Figure 1 Linear regression plot for TB antigen values and FPG.
