# Supplementary figures and images for: The association between diabetes status and latent-TB IGRA levels from a cross-sectional study in eastern China
Source: Front Cell Infect Microbiol. 2023 Jan 16;12:1057298. doi: 10.3389/fcimb.2022.1057298 (PMC9884689; doi:10.3389/fcimb.2022.1057298)

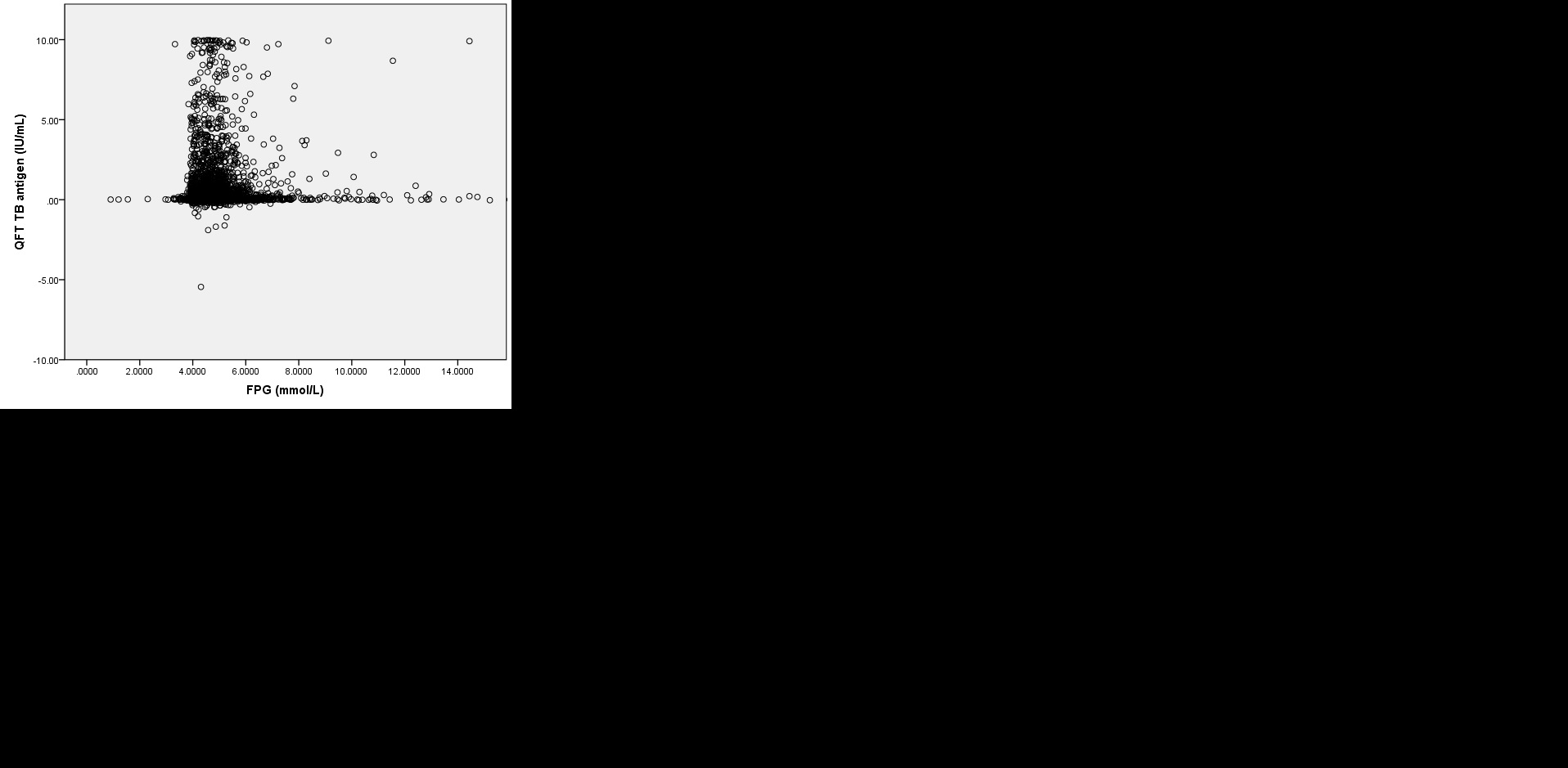

Supplement: Supplementary Figure 1 — Linear regression plot for TB antigen values and FPG. [file Image_1.jpeg]
